# Impaired Brain Astrocytic Glycolysis in Neuropathic Pain: Insights from Transcriptomic Profiling to Metabolic Intervention

**DOI:** 10.3390/ijms27198575

**Published:** 2026-09-25

**Authors:** Zhifeng Huang, Kailu Zou, Liqiong He, Malijiang Mulati, Qingwei Deng, Chunguang Yang, Changsheng Huang

**Affiliations:** 1 Department of Anesthesiology, Xiangya Hospital of Central South University, Changsha 410008, China238112350@csu.edu.cn (Q.D.);; 2 National Clinical Research Center for Geriatric Diseases, Xiangya Hospital of Central South University, Changsha 410008, China; 3 Hunan Province Clinical Research Center for Anesthesia and Perioperative Medicine, Changsha 410008, China

**Keywords:** neuropathic pain, astrocytes, metabolic reprogramming, glycolytic deficit, acetate

## Abstract

Neuropathic pain is a chronic disorder marked by sensory hypersensitivity and affective disturbances, yet current treatments often provide limited relief. Research on astrocytes in pain has focused largely on spinal mechanisms. Whether chronic peripheral nerve injury alters brain astrocytic metabolism remains unclear. Here, we isolated astrocytes from whole-brain preparations of adult mice after spared nerve injury (SNI) for transcriptomic profiling and identified coordinated suppression of astrocytic glycolysis, characterized by downregulation of genes (*Aldoc*, *Pfkp*, *Pfkm*, *Slc2a12*, and *Slc2a13*). Real-time Seahorse flux analysis showed reduced glycolytic activity and glycolytic capacity in SNI-derived astrocytes. We administered sodium acetate, an energy substrate preferentially utilized by astrocytes, intracerebroventricularly. Compared with vehicle treatment, acetate increased glycolytic activity and capacity in isolated astrocytes and partially improved pain-related behaviors in SNI mice. Our findings identify reduced glycolytic function in astrocytes isolated from whole-brain preparations after SNI. These findings support further investigation of astrocytic metabolism as a potential therapeutic target for chronic pain and its affective consequences.

## 1. Introduction

Neuropathic pain results from a lesion or disease of the somatosensory nervous system and is characterized by severe sensory hypersensitivity and a high prevalence of disabling affective comorbidities [1,2]. For decades, pain research has focused largely on neuronal hyperexcitability, ion channel dysfunction, and aberrant synaptic transmission [3]. However, therapies targeting these neuronal mechanisms often provide insufficient relief or cause intolerable central nervous system (CNS) side effects [4]. These limitations suggest that persistent pain involves both autonomous neuronal firing and disruption of the multicellular CNS environment.

Research has also expanded from isolated neural circuits to neuroglial interactions, identifying reactive astrocytes as important regulators of the development and maintenance of chronic pain [5,6]. Once regarded primarily as passive structural support cells, astrocytes are now recognized as active regulators of synaptic metabolism and function [7]. Following peripheral nerve injury, astrocytes undergo extensive morphological and biochemical remodeling, driving central sensitization through the secretion of neuroinflammatory mediators and the clearance of neurotransmitters [5,8]. These homeostatic functions may be particularly important in supraspinal circuits and affective processing. In these circuits, persistent pain imposes sustained metabolic demands on the neurovascular unit. Because astrocytic function is closely linked to intrinsic metabolic capacity, characterizing astrocytic metabolic remodeling may help explain how peripheral injury drives maladaptive changes in central neural networks [9,10].

Evidence from CNS disorders indicates that such astrocytic metabolic changes are stress-driven adaptations that actively influence disease progression, rather than merely passive consequences of cellular activation [11,12,13]. The effects on neuronal survival and circuit function can be beneficial or detrimental, depending on the nature and duration of the underlying neurological insult [11,14,15]. Targeted genetic or pharmacological manipulation of astrocytic pathways has demonstrated that altering glial glucose utilization can directly affect synaptic integrity and network excitability [11,13]. Astrocytic metabolism can support or disrupt neural homeostasis, depending on the disease context. This raises the question of how chronic pathological states may drive maladaptive metabolic remodeling that sustains circuit dysfunction.

Reactive astrogliosis in the spinal dorsal horn is well characterized as a contributor to local nociceptive sensitization. Persistent peripheral insults also impose sustained metabolic demands on supraspinal regions involved in sensory and affective processing [16]. However, whether brain astrocytes undergo coordinated metabolic dysfunction during chronic pain and whether this dysfunction contributes to associated affective comorbidities remain unclear. We hypothesized that persistent neuropathic pain places sustained bioenergetic stress on supraspinal astrocytes, inducing a maladaptive hypometabolic state and disrupting neuroglial metabolic coupling. To examine this hypothesis, we used a murine spared nerve injury (SNI) model. We combined magnetic-activated cell sorting of astrocytes from adult mice with high-throughput RNA sequencing and real-time Seahorse flux analysis. Astrocytes isolated from whole-brain preparations after SNI showed reduced expression of glycolysis-related genes and reduced glycolytic function. Compared with vehicle treatment, intracerebroventricular sodium acetate administration increased glycolytic activity and capacity in astrocytes isolated from SNI mice. Sodium acetate administration was also accompanied by changes in nociceptive measures and in forced swim, sucrose preference, and open-field test outcomes. These findings support further investigation of the functional significance of astrocytic metabolic alterations in neuropathic pain and of astrocyte-targeted metabolic restoration as a potential non-neuronal therapeutic strategy.

## 2. Results

### 2.1. Validation of the SNI Model and Quality Control of Astrocyte Transcriptomic Data

To investigate the astrocytic transcriptomic changes associated with neuropathic pain, we established a mouse SNI model (Figure 1A). Nociceptive sensitivity was evaluated by assessing the paw withdrawal mechanical threshold (PWMT) and paw withdrawal thermal latency (PWTL) at baseline and on postoperative days 1, 3, 7, and 14. Compared with sham controls, SNI mice showed progressive and significant reductions in both PWMT and PWTL that persisted through day 14 after surgery (Figure 1B,C). In SNI mice, the ipsilateral hind limb also exhibited lower PWMT and PWTL than the contralateral hind limb (Figure 1D,E), supporting successful model establishment.

On postoperative day 14, we isolated astrocyte-enriched populations from whole-brain preparations using magnetic-activated cell sorting (MACS) for transcriptomic profiling (Figure 1F). Immunofluorescence analysis showed a GFAP-positive cell proportion of 99%, calculated relative to the number of DAPI-positive nuclei (Figure 1G). Western blotting provided additional qualitative evidence of GFAP enrichment in the positively selected fraction (Figure S1). RNA-seq data showed prominent expression of astrocytic markers and lower expression of the non-astrocytic markers included in the panel (Figure 1H). All Q20 scores exceeded 96% (range, 96.72–97.62%), which is above the 95% threshold and consistent with low base-calling error rates (Figure 1I). In addition, genomic alignment yielded high mapping rates ranging from 94.47% to 98.05%, with a mean of 97.33% (Table S2). These results supported astrocyte enrichment and the suitability of the RNA-seq data for downstream analyses.

### 2.2. Altered Gene Expression Profiles in Mouse Brain Astrocytes Following SNI

Principal component analysis (PCA) revealed a clear separation between the SNI and Sham groups, indicating marked alterations in the gene expression profiles of brain astrocytes following SNI (Figure 2A). A total of 1491 differentially expressed genes (DEGs) were identified, including 440 upregulated and 1051 downregulated genes, based on the following criteria: adjusted *p* value ≤ 0.05 and |log_2_ fold change| ≥ 1 (Figure 2B,C).

Gene Ontology (GO) annotation of the DEGs was used to classify functional changes across cellular component (CC), molecular function (MF), and biological process (BP) categories (Figure 2D). Within the BP category, the enriched terms fell into three main functional themes. Ion homeostasis and transport terms included positive regulation of sodium ion export and cellular sodium efflux. Cellular organization and intercellular interaction terms included epithelial cilium movement, maintenance of cell–cell junctions, and neuron cell adhesion. Metabolic or immune-related terms included aspartate metabolism, regulation of bile acid metabolism, and neutrophil extravasation. Enrichment of the Na^+^/K^+^-ATPase complex, ion antiporter activity, and oxidoreductase activity in CC and MF suggested alterations in ATP-dependent membrane transport and redox-related enzymatic functions.

Consistent with the GO results, KEGG pathway enrichment analysis also highlighted metabolic and transport-related pathways (Figure 2E). Enriched pathways were mainly associated with amino acid and nitrogen metabolism, bile acid biosynthesis and secretion, vitamin/cofactor metabolism, ion-coupled transport processes, and neurotransmitter-related synaptic pathways. Notably, several pathways involved in sodium handling and acid–base balance were identified, underscoring the close coupling between ion transport and cellular energy metabolism.

Collectively, these findings reveal coordinated transcriptional alterations in energy-dependent ion transport, redox enzymes, and amino acid/bile acid metabolic pathways, suggesting broad changes in cellular bioenergetics and substrate utilization. This integrated transcriptional signature prompted further investigation of the metabolic alterations occurring in brain astrocytes during chronic neuropathic pain.

### 2.3. Metabolism-Related Transcriptional Changes in Astrocytes Following SNI

To characterize metabolic alterations in astrocytes after SNI, we identified 126 metabolism-related DEGs in the astrocyte transcriptomic profiling (Figure 3A, Table S3). Protein–protein interaction (PPI) network analysis of these metabolic genes revealed an interconnected network of their encoded proteins (Figure 3B). Proteins encoded by glycolysis-related genes were densely interconnected (Table S4). Genes related to mitochondrial function and oxidative metabolism, including regulators of mitochondrial biogenesis and redox homeostasis, formed a distinct, centrally connected module. This module correlated glycolysis with downstream energy metabolism within the network. Additionally, metabolic pathways related to amino acid metabolism, one-carbon metabolism, and metabolite transport were organized into interconnected subnetworks, suggesting interactions among energy production, biosynthetic pathways, and substrate transport. Together, these network features suggest that under neuropathic pain conditions, astrocytic metabolic pathways undergo coordinated yet pathway-specific reorganization.

Consistent with the network-level organization observed in the PPI analysis, pathway enrichment analysis of metabolism-related DEGs revealed significant enrichment of multiple core metabolic pathways (Figure 3C). These included biosynthesis of amino acids, biosynthesis of unsaturated fatty acids, carbon metabolism, fatty acid metabolism, glycolysis/gluconeogenesis, and the pentose phosphate pathway. Several of these enriched pathways converged on glucose utilization, lipid metabolism, and redox balance, suggesting that astrocytic metabolic alterations reflect coordinated remodeling of interconnected metabolic systems rather than changes confined to individual pathways.

### 2.4. Reduced Glycolysis-Related Gene Expression and Glycolytic Capacity in Astrocytes Following SNI

To characterize the metabolic changes associated with astrocytic reprogramming after SNI, we focused on genes involved in glucose transport and glycolysis. We observed significant downregulation of several glycolysis-related genes in the SNI group, including *Aldoc*, *Pfkp*, *Pfkm*, and *Slc2a12*, together with a reduced expression of *Slc2a13*, which encodes the proton-coupled myo-inositol transporter HMIT (Figure 4A–E). Gene set enrichment analysis further confirmed a suppression of the glycolysis pathway in SNI astrocytes (negative enrichment, NES < 0), supporting coordinated suppression of glycolysis-related gene expression under neuropathic pain conditions (Figure 4F).

We assessed the expression of these genes by quantitative PCR (qPCR) in brain astrocytes isolated from mice 14 days after SNI. The expression levels of *Aldoc*, *Pfkp*, *Pfkm*, *Slc2a12*, and *Slc2a13* were significantly reduced, confirming the sequencing results (Figure 4G). To assess whether these molecular changes were accompanied by functional metabolic impairment, we measured the extracellular acidification rate (ECAR) using Seahorse analysis. In line with the above results, astrocytes from SNI mice exhibited reduced glycolytic activity and glycolytic capacity (Figure 4H–J).

Together, these results identify reduced expression of selected glycolysis-related genes and reduced glycolytic activity and capacity in the brain astrocytes after SNI. Given the reliance of astrocytes on glycolysis for ATP generation and lactate-mediated metabolic support to neurons, impaired glycolytic capacity may contribute to disrupted astrocyte–neuron metabolic coupling during chronic neuropathic pain.

### 2.5. Acetate Administration Enhances Astrocytic Glycolytic Capacity and Ameliorates Pain-Associated Behaviors

Given the reduced glycolytic activity and capacity observed in astrocytes after SNI, we next examined whether metabolic supplementation could improve astrocytic glycolytic function and alleviate pain-associated sensory and affective behavioral changes. We selected sodium acetate, an astrocyte-preferential energy substrate that can support astrocytic metabolism. SNI mice received intracerebroventricular infusion of sodium acetate, and astrocytic glycolysis was assessed according to the timeline shown in Figure 5A. Seahorse ECAR analysis showed higher glycolytic activity and glycolytic capacity in astrocytes isolated from acetate-treated SNI mice than in those isolated from vehicle-treated SNI mice (Figure 5B–D).

Next, we evaluated pain-related behaviors according to the experimental timeline in Figure 5E. Compared with vehicle-treated SNI mice, acetate-treated SNI mice had significantly higher PWMT and PWTL, indicating attenuation of mechanical allodynia and thermal hyperalgesia (Figure 5F,G). Acetate treatment also significantly reduced immobility time in the forced swim test and increased sucrose preference in SNI mice (Figure 5H,I). In the open-field test, acetate-treated SNI mice spent significantly more time in the center zone than vehicle-treated SNI mice (Figure 5J). Both vehicle- and acetate-treated SNI mice exhibited significantly lower PWMT and PWTL, longer forced-swim immobility, and less center-zone time than their respective sham controls. These comparisons indicated partial behavioral improvement following acetate treatment in SNI mice. No significant between-group differences were detected in total distance traveled or locomotor speed across the four groups (Figure S2). Together, the glycolytic and behavioral responses to acetate supported a functional link between astrocytic metabolic impairment and the maintenance of chronic pain-related behaviors.

## 3. Discussion

Neuropathic pain is a debilitating chronic condition involving maladaptive central plasticity and frequent affective comorbidities, and current therapies remain insufficient [1,2,17,18]. Astrocytes regulate pain chronification by modulating synaptic function and neuroimmune signaling [6,19]. Our study adds a metabolic dimension to this framework by identifying impaired glycolysis in astrocytes isolated from whole-brain preparations after SNI. Transcriptomic profiling and targeted gene-expression analyses revealed reduced expression of multiple glycolysis-related genes, accompanied by decreases in basal glycolysis and glycolytic capacity. These findings provide an experimental basis for investigating the contribution of astrocytic metabolic dysfunction to pain persistence and a rationale for exploring metabolic approaches to intervention.

Research on reactive astrogliosis in chronic pain has focused largely on spinal nociceptive sensitization and neuroinflammatory signaling [20,21]. Neuropathic pain also involves maladaptive remodeling of affective, cognitive, and motivational brain networks [22,23]. These brain changes warrant examining astrocytic responses beyond the spinal cord. Our profiling data reveal an aggregate transcriptomic signature in astrocytes isolated from whole-brain preparations after prolonged peripheral nerve injury. These findings provide an experimental basis for determining which brain regions and astrocyte populations contribute to the observed metabolic alterations and for investigating how these changes relate to sensory and affective dysfunction.

The reduction in glycolytic function observed in astrocytes isolated from whole-brain preparations raises questions regarding the anatomical and temporal context of astrocytic metabolic responses. Previous work indicates that spinal cord astrocytes increase glycolysis and accelerate lactate production to support local nociceptive amplification during acute sensitization [24]. Differences in anatomical location and disease phase may contribute to these contrasting findings. One hypothesis is that astrocytes exposed to persistent stress may enter a state of reduced glycolytic capacity. This hypothesis is consistent with the cell-autonomous metabolic response to inflammatory stimulation reported in vitro by Robb et al. [25]. Their work established that 3 h of lipopolysaccharide exposure increased astrocytic glycolysis, whereas 24 h exposure conversely reduced glycolytic capacity and glucose transport machinery. Whether a comparable temporal transition occurs after SNI and which brain regions contribute to the observed glycolytic reduction remain unknown. At a functional level, reduced astrocytic glycolytic capacity could limit metabolic support to higher-order neural networks and thereby affect synaptic homeostasis and circuit function, a possibility that warrants direct investigation.

Astrocytic glycolytic impairment may also be relevant to affective comorbidities in neuropathic pain. Experimental disruption of astrocytic LDHA-dependent lactate homeostasis has been shown to alter neuronal excitability and promote depressive-like behaviors in mice [26], providing a rationale for considering metabolic links between persistent nociception and affective disturbances [27]. Related work has implicated impaired lactate release from dorsal hippocampal CA1 astrocytes in nociceptive sensitization and comorbid memory deficits after SNI [28]. Together with our transcriptomic and functional findings, these observations support the hypothesis that astrocytic metabolic dysfunction may contribute to both sensory and affective dysfunction in chronic pain. Whether these behavioral dimensions share a causal metabolic mechanism and whether common or distinct regional astrocyte populations are involved remain to be established.

The partial behavioral improvement following sodium acetate administration supports a functional link between astrocytic metabolism and neuropathic pain phenotypes. Exogenous acetate is preferentially taken up by astrocytes [29,30], where it promotes glycolysis and neurotransmitter cycling [31]. Our finding that central acetate administration increased glycolytic activity and capacity in astrocytes while attenuating both mechanical hypersensitivity and depressive-like behaviors suggests a potential link between astrocytic metabolic function and pain-related behavioral outcomes. However, preferential acetate utilization does not establish astrocyte-specific action under the present experimental conditions. Acetate may also exert metabolic effects beyond glycolysis, including potential effects on anaplerosis and redox homeostasis. Because astrocytic glycolysis was not selectively manipulated, the behavioral improvements cannot be attributed specifically to changes in this pathway. Further studies should clarify the cellular and metabolic mechanisms underlying the effects of acetate and identify the brain regions involved.

Although our analyses identify reduced glycolytic function in astrocytes isolated from whole-brain preparations, several important questions remain to be explored. Whole-brain sampling cannot resolve regional or hemispheric heterogeneity, and differences in the relative representation of astrocytes from different brain regions within the isolated populations may contribute to the bulk transcriptomic signature. Spatially resolved transcriptomic analyses, complemented by targeted functional validation, will be needed to determine the anatomical distribution of these changes across sensory and affective brain regions. Longitudinal analyses are also required to define the temporal evolution of astrocytic metabolic alterations following peripheral nerve injury. The open-field locomotor measurements do not fully exclude effects on sedation or reward processing as alternative explanations for the behavioral findings. Furthermore, only male mice were studied; therefore, potential sex differences were not assessed, and the applicability of our findings to females remains to be established. Finally, although real-time flux analyses confirm impaired glycolytic function in isolated astrocytes, high-glucose, serum-containing culture conditions may influence this phenotype. In vivo measurements of cerebral glucose metabolism and local neuron–glia metabolic exchange will be needed to assess the relevance of these findings and guide future metabolic interventions.

## 4. Materials and Methods

### 4.1. Animals

Adult male C57BL/6 mice (6–8 weeks; Beijing Vital River Laboratory Animal Technology, Beijing, China) were group-housed in individually ventilated cages under standard controlled conditions (12-h light/dark cycle, 22–25 °C, 50–60% humidity) with ad libitum access to food and water. Mice were randomly assigned to the experimental groups using a random number generator. All animal procedures were approved by the Institutional Ethics Committee of Xiangya Hospital, Central South University, and strictly adhered to NIH ethical guidelines.

### 4.2. Establishment of the Neuropathic Pain Model

Neuropathic pain was induced by spared nerve injury (SNI) as described previously [32,33]. The left sciatic nerve and its trifurcation were exposed under isoflurane anesthesia. The common peroneal and tibial nerves were tightly ligated with 8-0 nylon sutures and transected distally (2–4 mm excised) to prevent regeneration, while the sural nerve was left intact. Sham-operated mice underwent the same surgical exposure without nerve ligation.

### 4.3. Behavioral Tests

Nociceptive sensitization assays: Following a 30 min acclimation, the paw withdrawal mechanical threshold (PWMT) of the mice’s hind paws was determined using von Frey filaments via the up–down method [34]. PWMT was defined as the minimum force eliciting a withdrawal response across three consecutive trials. Paw withdrawal thermal latency (PWTL) was measured using a plantar test apparatus (Tes7370, Ugo Basile, Comerio, Italy) by directing a focused radiant heat source to the plantar surface [35]. A 25-s thermal cutoff was applied to prevent tissue damage. For both assays, three measurements were averaged with 5 min inter-trial intervals to prevent sensitization. Affective assays: (1) Depressive-like/stress-coping behavior was evaluated using the forced swim test (FST) [36]; mice swam in a water-filled cylinder (15 cm depth, 24 ± 1 °C) for 6 min, and immobility time was quantified during the final 4 min. (2) Anhedonia-like behavior was assessed using the sucrose preference test (SPT) [37]; mice were singly housed and habituated to a two-bottle choice paradigm (2% sucrose vs. water) for 48 h, and sucrose preference was calculated as sucrose consumption (g)/total fluid consumption (g) × 100%. (3) Anxiety-like behavior was assessed via the open-field test (OFT) [38]; mice freely explored a square arena for 10 min under dim lighting, and the total time spent in the center zone was recorded by a video-tracking system. All behavioral evaluations were conducted by investigators blinded to group allocations.

### 4.4. Isolation of Astrocytes from the Mouse Brain

Astrocytes were isolated from the whole brains of adult mice using magnetic-activated cell sorting (MACS). Brains were enzymatically and mechanically dissociated using the Adult Brain Dissociation Kit and gentleMACS Octo Dissociator (Miltenyi Biotec, Bergisch Gladbach, Germany). Homogenates were filtered (70-μm) and subjected to sequential myelin and erythrocyte removal. The resulting cell suspension was Fc-blocked and incubated with Anti-ACSA-2 MicroBeads (Miltenyi Biotec, Bergisch Gladbach, Germany) for astrocyte enrichment via LS columns (Miltenyi Biotec, Bergisch Gladbach, Germany), following established protocols [39,40].

### 4.5. RNA Sequencing and Bioinformatic Analyses

Four pooled samples per group were initially submitted for RNA sequencing, with each pool comprising material from three mice in the same group (12 mice per group). One pooled sample from each group was excluded because laboratory records documented contamination. Differential expression analysis used the remaining three pooled samples per group, with each pool treated as one biological observation. Total RNA integrity was assessed via an Agilent Bioanalyzer before generating strand-specific poly-A libraries for 150 bp paired-end sequencing on an Illumina NovaSeq 6000 platform (Shanghai Biotechnology Corporation, Shanghai, China). Raw reads were quality-filtered using Fastp (v0.23.2), aligned to the mouse reference genome (GRCm38/mm10) via STAR (v2.7.10b), and quantified with StringTie (v2.2.1). Differentially expressed genes (DEGs) were identified using DESeq2 (v1.38.3; adjusted *p* value ≤ 0.05, |log_2_FC| ≥ 1) and subjected to GO/KEGG functional enrichment via the clusterProfiler package (v4.8.1) [41,42]. Protein–protein interaction (PPI) networks were constructed using the STRING database (confidence score ≥ 0.4) and visualized in Cytoscape (version 3.10.0), where node degree was calculated to identify critical hub proteins within metabolic modules [43].

### 4.6. Reverse Transcription Quantitative PCR

Total RNA was extracted using the Ultrapure RNA Kit (CoWin Biotech, Taizhou, China) and reverse-transcribed (500 ng) using the EasyScript cDNA Synthesis SuperMix (TransGen Biotech, Beijing, China). Quantitative PCR was performed with 96-well PCR plates (EIKON HEALTH, Dallas, TX, USA) and Universal SYBR qPCR Master Mix (Kunming YoungGen Biotech, Kunming, China) on a QuantStudio 5 system (Applied Biosystems, Waltham, MA, USA). Relative mRNA expression was calculated via the 2^−ΔΔCt^ method and normalized to an endogenous reference gene (primer sequences listed in Table S1). Six biological samples were analyzed per group, each obtained from a different mouse. Each sample was assayed in three technical replicates, which were summarized within each biological sample before statistical analysis.

### 4.7. Western Blot

Astrocytes were lysed in RIPA buffer containing protease inhibitors (NCM Biotech, Suzhou, China), and protein concentrations were determined via BCA assay. Equal protein amounts were resolved on 4–12% SDS-PAGE precast gels (ACE, Shanghai, China) and transferred to PVDF membranes. Following a 30 min block at room temperature (BIOLIGO Fast Blocking, Shanghai, China), membranes were incubated overnight at 4 °C with primary antibodies: mouse anti-GFAP (1:1000; Cat# 250027, zenbio, Chengdu, China) and rabbit anti-GAPDH (1:20,000; Proteintech, Wuhan, China). After TBST washes, membranes were incubated with species-specific HRP-conjugated secondary antibodies for 1 h at room temperature. Immunoreactive bands were visualized using enhanced chemiluminescence (KeygenBioTECH, Nanjing, China).

### 4.8. Astrocyte Culture and Immunofluorescence

Primary astrocytes were maintained in high-glucose DMEM (Cat# U21-265B, YOBIBIO, Shanghai, China) supplemented with 5% fetal bovine serum (Cat# F814-100, BaiDi Biotechnology Co., Ltd., Guangzhou, China), 1% penicillin–streptomycin, and 1% astrocyte growth supplement (Abiowell, Changsha, China), at 37 °C in 5% CO_2_. For immunofluorescence, cells seeded on coverslips were fixed with 4% paraformaldehyde, permeabilized with 0.3% Triton X-100, and blocked with 5% normal serum. Cells were incubated overnight at 4 °C with a primary antibody against GFAP, followed by incubation with an Alexa Fluor-conjugated secondary antibody (1:200, Abcam, Cambridge, UK) for 1 h at room temperature. Nuclei were counterstained with DAPI. Coverslips were mounted with anti-fade medium and imaged via confocal microscopy. For immunofluorescence assessment, MACS-isolated cells were cultured for 4–5 days without passaging before fixation. Three independent preparations were examined, each obtained from one mouse, with three microscopic fields analyzed per preparation. For each preparation, 70–100 cells were counted per field of view. In each field, the GFAP-positive cell proportion was calculated as the number of GFAP-positive cells associated with DAPI-stained nuclei divided by the total number of DAPI-positive nuclei, multiplied by 100. Quantification was summarized at the mouse level, and the reported mean was calculated across the three independent preparations (n = 3).

### 4.9. Extracellular Acidification Rate Measurement

Following isolation, astrocytes were cultured for 48 h in high-glucose DMEM supplemented with 5% fetal bovine serum, as described in Section 4.8. Cells were then seeded onto Seahorse XFe96 (Agilent Technologies, Santa Clara, CA, USA) assay plates at 2 × 10^4^ cells per well and cultured in the same medium for an additional 12 h before assay preparation. Cells were not subjected to serial passaging. The extracellular acidification rate (ECAR) of primary astrocytes isolated from adult mice was evaluated using a Seahorse XFe96 Extracellular Flux Analyzer (Agilent Technologies, New Brunswick, NJ, USA) according to the manufacturer’s standard protocol. Briefly, the culture medium was replaced with pre-warmed Seahorse XF (Agilent Technologies, Santa Clara, CA, USA) assay medium (phenol red-free DMEM supplemented with L-glutamine, pH 7.4), and the cells were equilibrated in a 37 °C non-CO_2_ incubator for 1 h prior to measurement. Following sensor cartridge calibration, metabolic modulators were sequentially injected at the following final concentrations: D-glucose (10 mM) to stimulate basal glycolysis, oligomycin (2 μM) to determine maximal glycolytic capacity, and 2-deoxyglucose (2-DG, 50 mM) to shut down glycolysis and measure background non-glycolytic acidification. Real-time metabolic data were captured and analyzed using Wave software (version 2.4, Agilent Technologies, Santa Clara, CA, USA). ECAR values were reported in mpH/min with normalization to cell number. Six biological samples per group were analyzed, each derived from a different mouse and measured in three technical wells. Technical replicates were summarized within each biological sample before statistical analysis.

### 4.10. Intra-Cerebroventricular Injection

Under anesthesia, guide cannulas (Shanghai Yuyan Scientific Instruments Co., Ltd., Shanghai, China) were stereotaxically implanted into the lateral ventricle (coordinates from bregma: AP −0.5 mm, ML 1.1 mm, DV 2.3 mm). Following a one-week recovery, mice received daily intracerebroventricular (i.c.v.) microinjections of sodium acetate (120 mM dissolved in sterile aCSF; 2 μL/mouse at 0.25 μL/min) or an equal volume of vehicle (aCSF) for 7 consecutive days. Intracerebroventricular microinjections were performed via the implanted cannula using a microinfusion pump (RWD Life Science, Shenzhen, China).

### 4.11. Statistical Analysis

Data are expressed as mean ± standard deviation and were analyzed using GraphPad Prism 9.0. Seahorse comparisons between independent groups were performed using two-tailed unpaired Student’s *t*-tests. Behavioral outcomes measured at a single time point were compared among the Sham + Vehicle, Sham + Acetate, SNI + Vehicle, and SNI + Acetate groups. These outcomes included forced-swim immobility, sucrose preference, open-field center-zone time, total distance traveled, and locomotor speed. Comparisons used one-way ANOVA followed by Tukey’s multiple-comparison test for all pairwise group comparisons. Longitudinal behavioral data were analyzed using two-way repeated-measures ANOVA, and multi-gene qPCR data were analyzed using two-way ANOVA, followed in each case by Tukey’s multiple-comparison test. Technical replicates were summarized within each biological sample before statistical analysis. Sample sizes were selected with reference to previous basic research studies; no formal a priori power calculation was performed. Statistical significance was defined as *p* < 0.05.

## 5. Conclusions

In summary, SNI was associated with reduced glycolysis-related gene expression and glycolytic function in astrocytes isolated from whole-brain preparations. Central acetate administration increased astrocytic glycolytic measures and alleviated sensory and affective pain-related behaviors in SNI mice. These findings support further investigation of astrocytic metabolism as a potential therapeutic target, while the cellular and metabolic mechanisms underlying the effects of acetate remain to be established.

## Figures and Tables

**Figure 1:**
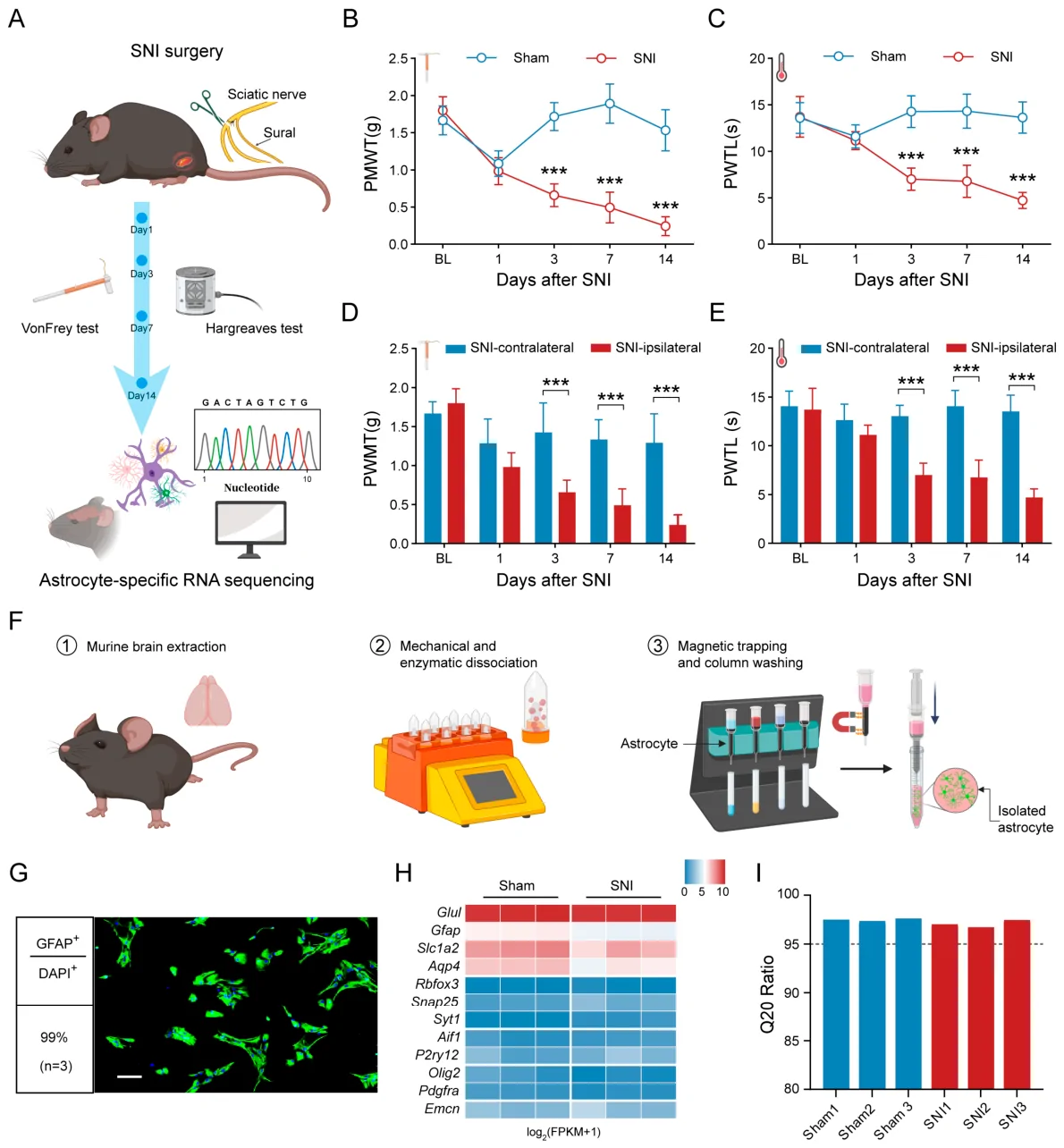
Validation of the SNI model and quality control of astrocyte transcriptomic profiling: (**A**) Schematic illustration of the SNI model, behavioral assessment timeline, and astrocyte-specific RNA sequencing workflow. (**B**,**C**) Paw withdrawal mechanical threshold (PWMT) and paw withdrawal thermal latency (PWTL) in the injured ipsilateral hind limb of mice after sham or SNI surgery. *** *p* < 0.001, n = 8, two-way repeated-measures ANOVA followed by Tukey’s multiple-comparison test. (**D**,**E**) PWMT and PWTL in the ipsilateral and contralateral hind limbs of mice after SNI surgery. *** *p* < 0.001, n = 8, two-way repeated-measures ANOVA followed by Tukey’s multiple-comparison test. (**F**) Workflow for astrocyte isolation and enrichment from adult mouse whole-brain preparations using magnetic-activated cell sorting (MACS). (**G**) Representative immunofluorescence images of the isolated cells (GFAP, green; DAPI, blue). Scale bar = 100 μm. (**H**) Heatmap of selected cell-type-associated marker genes in the sham and SNI groups (three RNA-seq samples per group). Markers include astrocytic (*Glul*, *Gfap*, *Slc1a2*, and *Aqp4*), neuronal (*Rbfox3*, *Snap25*, and *Syt1*), microglial (*Aif1* and *P2ry12*), oligodendrocyte lineage (*Olig2* and *Pdgfra*), and endothelial-associated (*Emcn*) genes. (**I**) Sequencing quality (Q20) scores for the sham and SNI groups (n = 3 per group).

**Figure 2:**
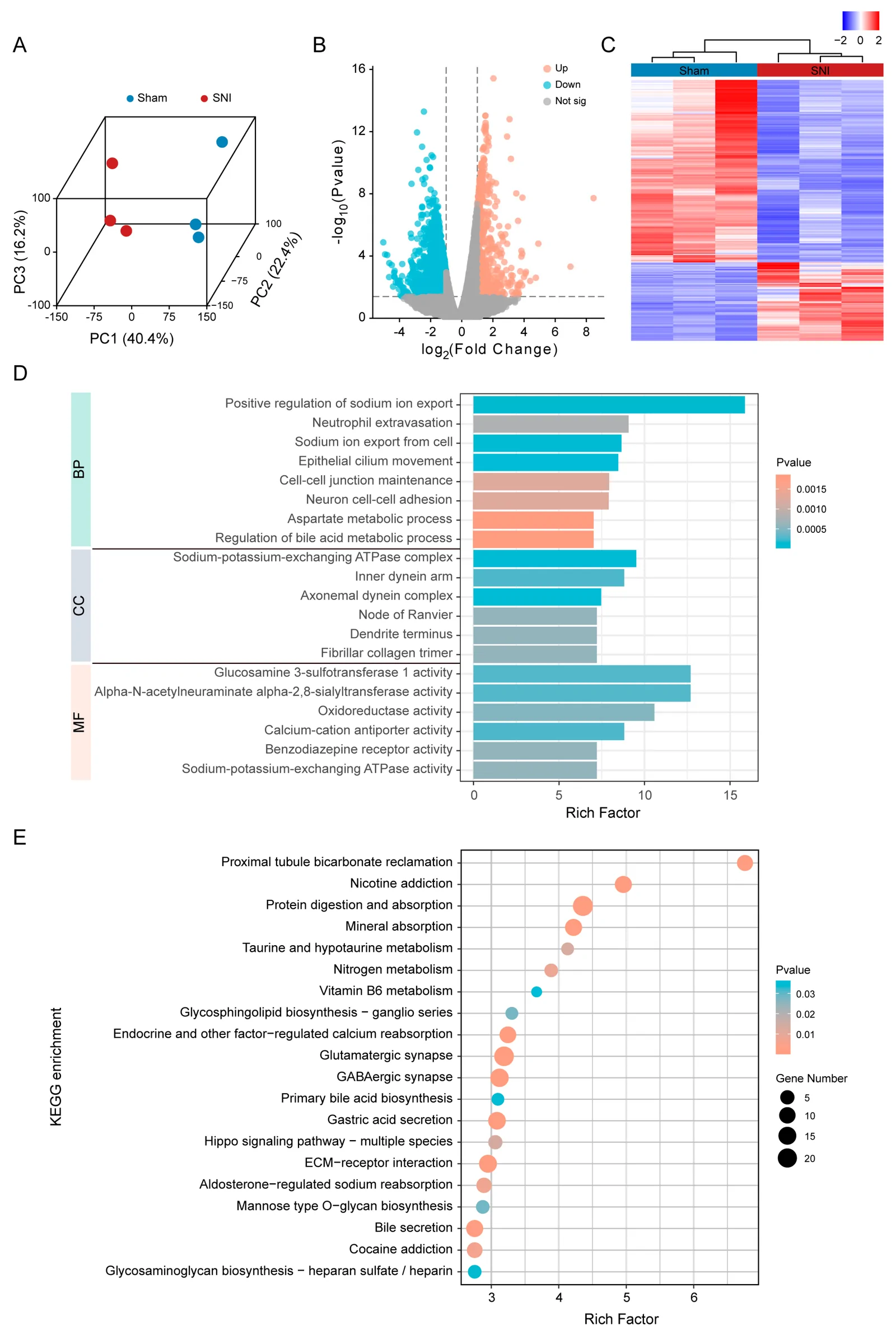
Transcriptomic profiling of brain astrocytes following SNI: (**A**) Principal component analysis (PCA) of astrocyte transcriptomes showing separation between Sham and SNI groups. (**B**) Volcano plot of differentially expressed genes (DEGs) in SNI versus sham astrocytes (adjusted *p* ≤ 0.05, |log_2_ fold change| ≥ 1). (**C**) Heatmap of DEGs illustrating relative expression patterns across groups. (**D**) Gene Ontology (GO) enrichment analysis of DEGs (biological process, cellular component, and molecular function). (**E**) Kyoto Encyclopedia of Genes and Genomes (KEGG) enrichment analysis of DEGs.

**Figure 3:**
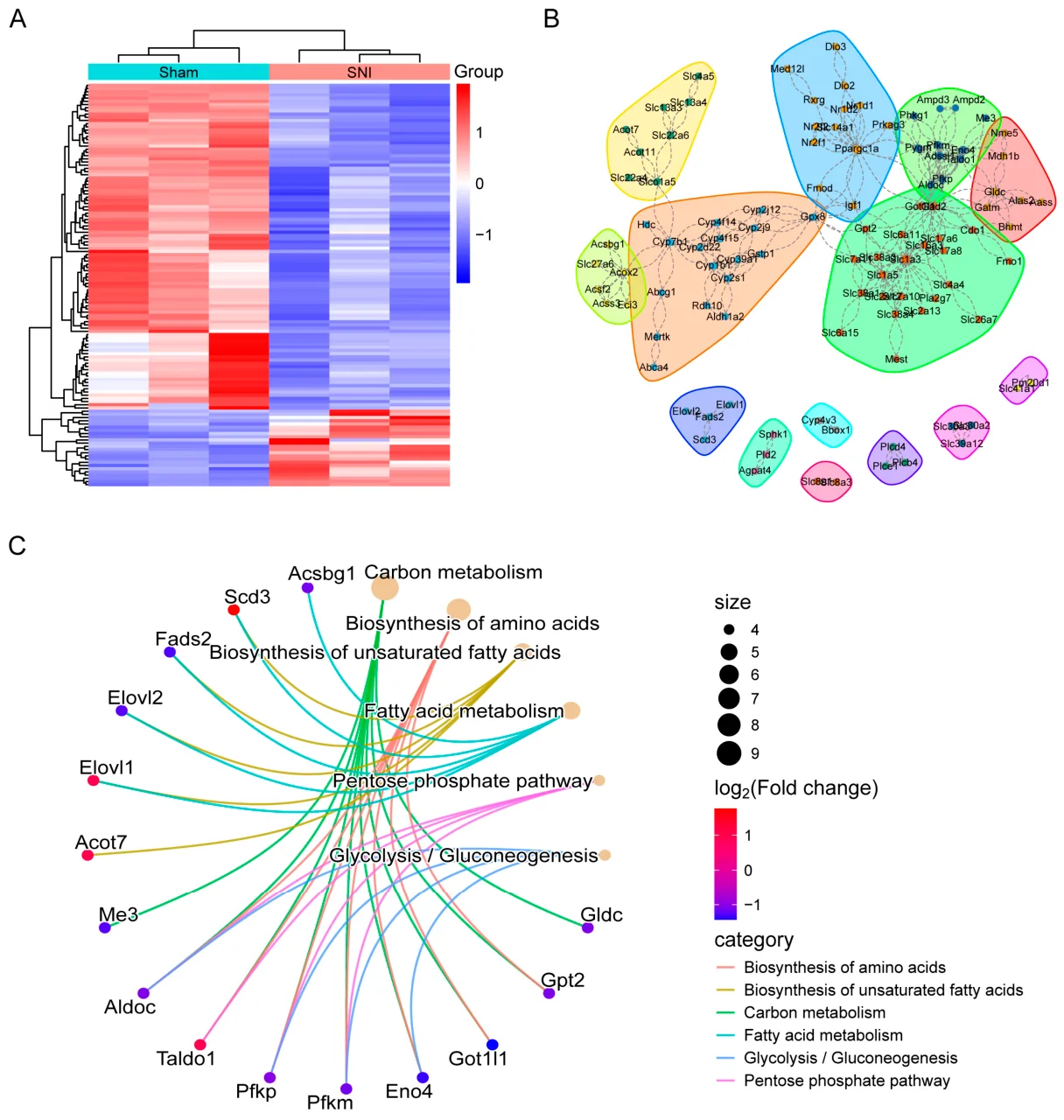
Metabolism-related transcriptional alterations in astrocytes isolated from whole-brain preparations after SNI: (**A**) Heatmap of 126 metabolism-related DEGs identified from astrocyte-specific transcriptomic profiling in Sham and SNI groups. (**B**) Protein–protein interaction (PPI) network constructed from the metabolism-related DEGs, illustrating overall connectivity and modular organization. Nodes represent gene products, and edges denote known or predicted functional interactions; colored regions indicate major network modules. (**C**) Pathway enrichment and gene–pathway association network for metabolism-related DEGs. Gene nodes are colored by log_2_(fold change), and node size reflects connectivity.

**Figure 4:**
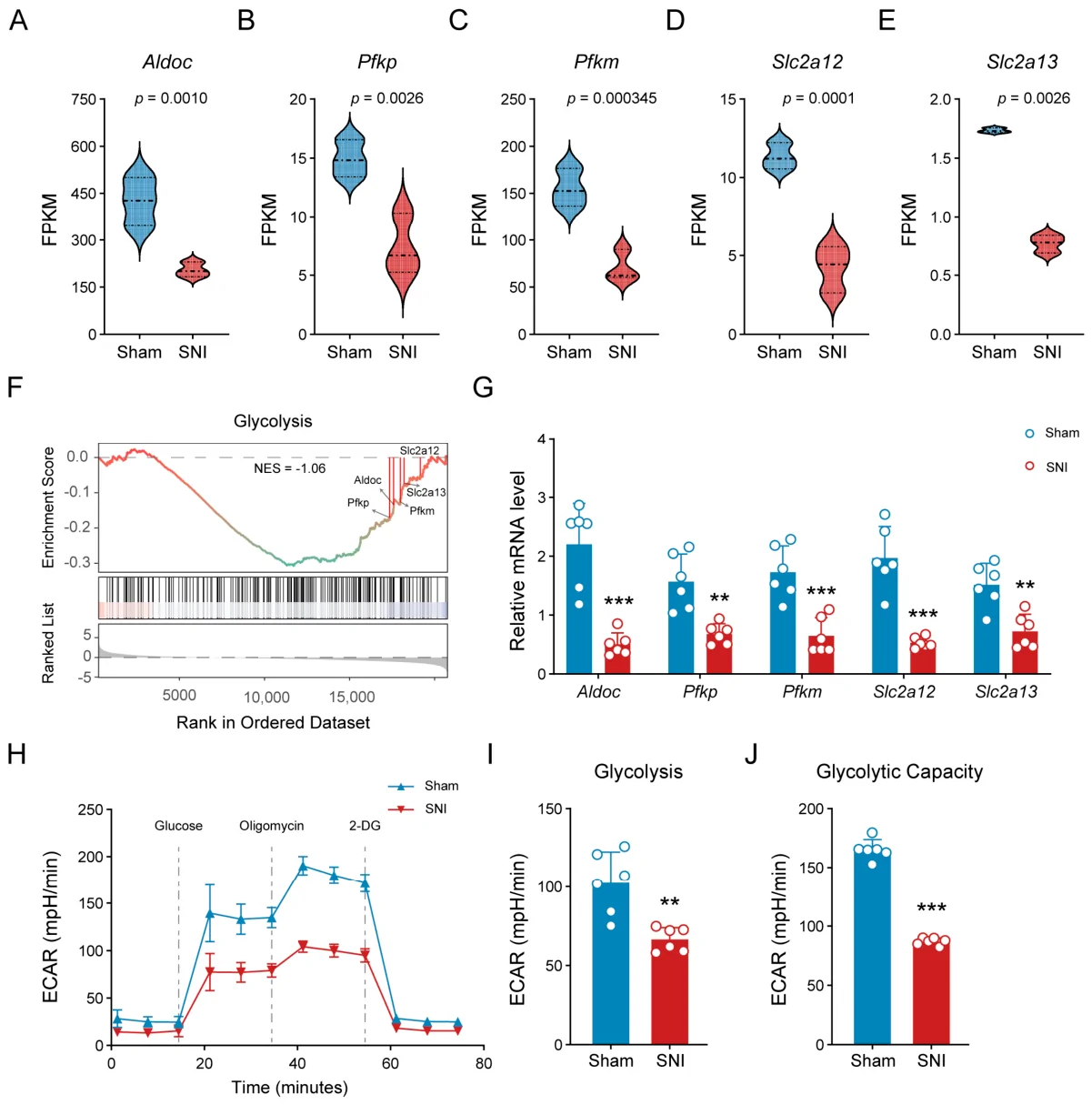
Reduced glycolysis-related gene expression and glycolytic function in isolated astrocytes after SNI: (**A**–**E**) Violin plots of RNA-seq expression levels (FPKM) of *Aldoc*, *Pfkp*, *Pfkm*, *Slc2a12*, and *Slc2a13* in astrocytes from Sham and SNI mice. (**F**) Gene set enrichment analysis (GSEA) demonstrating negative enrichment of the glycolysis pathway in SNI astrocytes (NES < 0). (**G**) qPCR validation of gene expression in brain astrocytes at 14 days after sham or SNI surgery. ** *p* < 0.01, *** *p* < 0.001, n = 6, two-way ANOVA followed by Tukey’s multiple-comparison test. (**H**–**J**) Extracellular acidification rate (ECAR) analysis with quantification of glycolysis and glycolytic capacity in the Sham and SNI groups. ** *p* < 0.01, *** *p* < 0.001, n = 6, two-tailed unpaired Student’s *t*-test.

**Figure 5:**
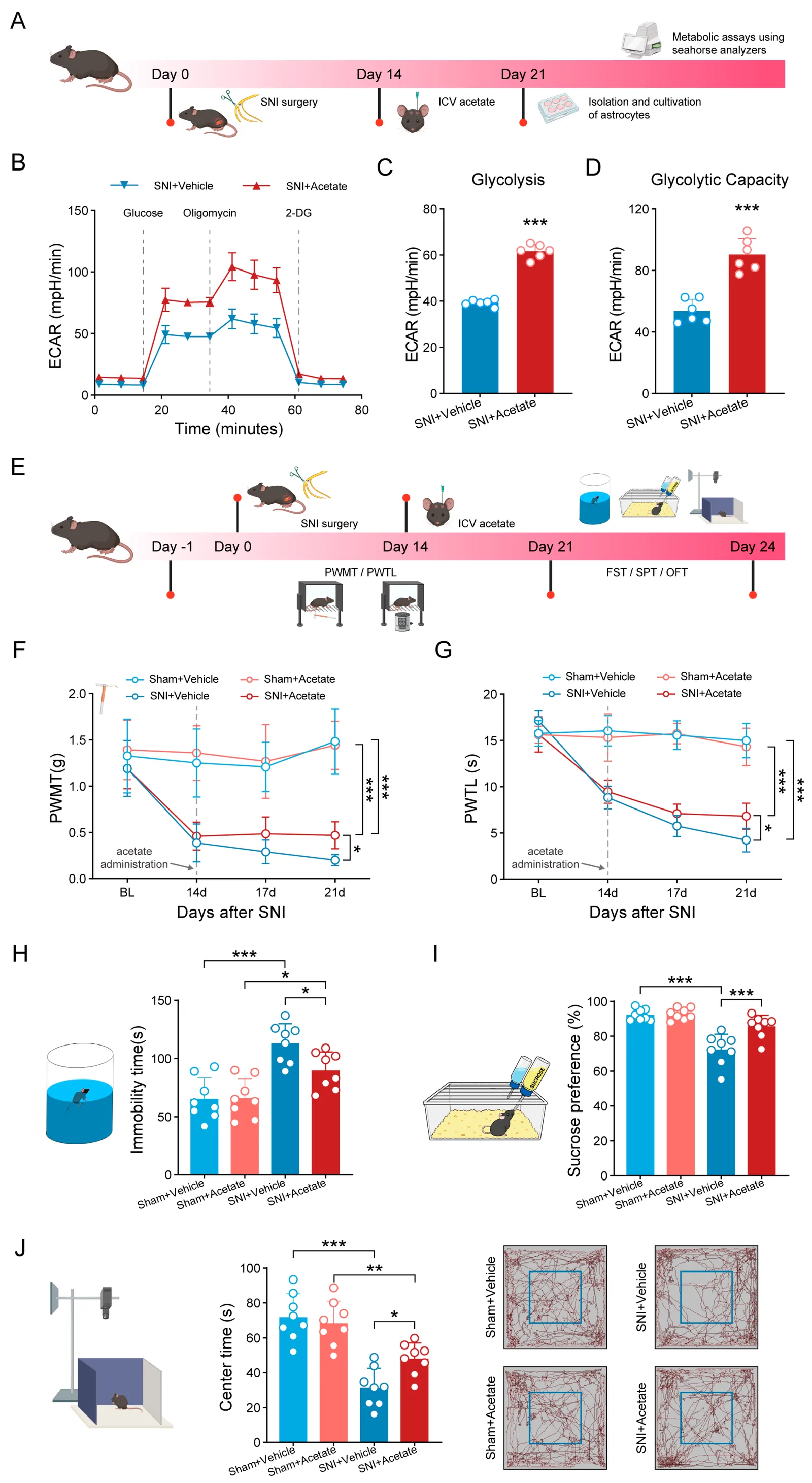
Effects of acetate administration on glycolytic and behavioral measures in SNI mice: (**A**) Experimental timeline for the Seahorse metabolic analysis. (**B**–**D**) Extracellular acidification rate (ECAR) analysis and quantification of glycolysis and glycolytic capacity in astrocytes isolated from acetate- or vehicle-treated SNI mice. *** *p* < 0.001, n = 6, two-tailed unpaired Student’s *t*-test. (**E**) Experimental design and timeline for behavioral testing. (**F**,**G**) PWMT and PWTL after 7 days of daily intracerebroventricular (i.c.v.) administration of artificial cerebrospinal fluid (aCSF; control) or sodium acetate, starting 14 days after SNI. * *p* < 0.05, *** *p* < 0.001, n = 8, two-way ANOVA with repeated measures followed by Tukey’s multiple-comparison test. (**H**–**J**) Immobility time in the forced swim test (FST), sucrose preference in the sucrose preference test (SPT), and time spent in the center zone in the open-field test (OFT) in the Sham + Vehicle, Sham + Acetate, SNI + Vehicle, and SNI + Acetate groups. * *p* < 0.05, ** *p* < 0.01, *** *p* < 0.001, n = 8, one-way ANOVA followed by Tukey’s multiple-comparison test for all pairwise group comparisons.

## Data Availability

The data supporting the findings of this study are available from the corresponding author upon reasonable request. The data are not publicly available at this time because they are also being used in ongoing research that has not yet been published.

## Supplementary Materials

The following supporting information can be downloaded at https://www.mdpi.com/article/10.3390/ijms27198575/s1.

## Abbreviations

The following abbreviations are used in this manuscript:

SNI	spared nerve injury
CNS	central nervous system
PWMT	paw withdrawal mechanical threshold
PWTL	paw withdrawal thermal latency
FST	forced swim test
SPT	sucrose preference test
OFT	open-field test
MACS	magnetic-activated cell sorting
DEGs	differentially expressed genes
PPI	protein–protein interaction
PCA	principal component analysis
GO	Gene Ontology
BP	biological process
CC	cellular component
MF	molecular function
KEGG	Kyoto Encyclopedia of Genes and Genomes
GSEA	gene set enrichment analysis
ECAR	extracellular acidification rate
aCSF	artificial cerebrospinal fluid
