# Supplementary material for: Impaired Brain Astrocytic Glycolysis in Neuropathic Pain: Insights from Transcriptomic Profiling to Metabolic Intervention
Source: Int J Mol Sci. 2026 Sep 25;27(19):8575. doi: 10.3390/ijms27198575 (PMC13665073; doi:10.3390/ijms27198575)
Supplement: Supplementary file 1 [file ijms-27-08575-s001.zip › ijms-4583871-supplementary.pdf]

Supplementary Materials

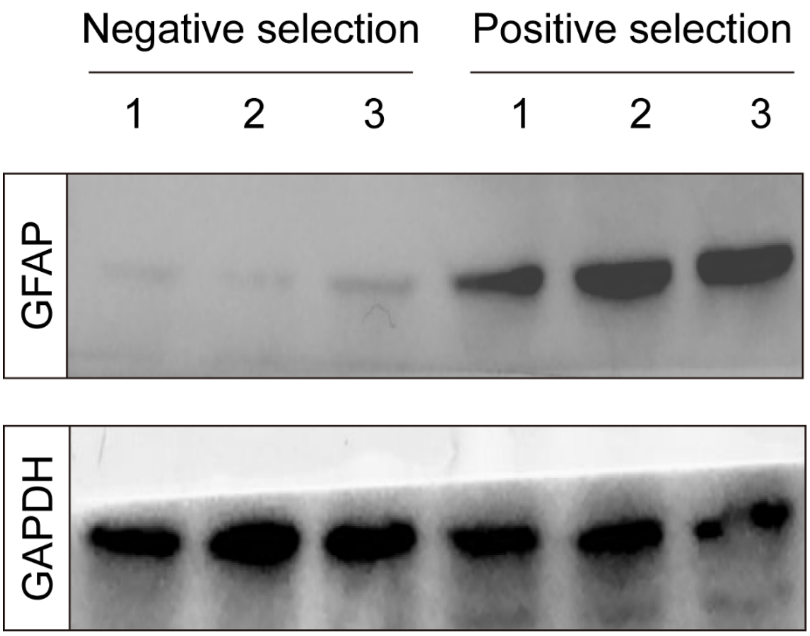

Figure S1: Qualitative assessment of GFAP enrichment following MACS isolation. Western blot validation of MACS separation (n = 3).

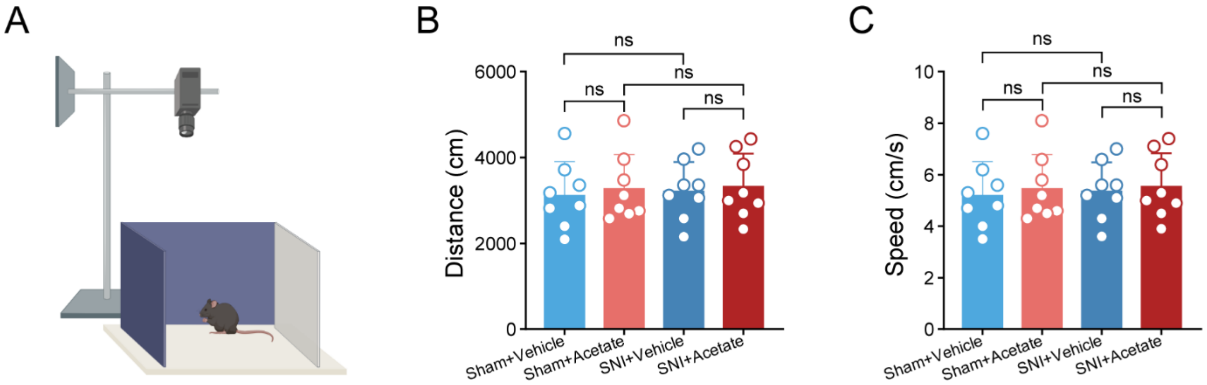

Figure S2: Effects of acetate administration on total distance traveled and locomotor speed in the open field test in sham and SNI mice. (A) Schematic illustration of the open field test. (B, C) Total distance traveled (B) and locomotor speed (C) in the Sham + Vehicle, Sham + Acetate, SNI + Vehicle, and SNI + Acetate groups. Data are presented as mean ± SD. Each symbol represents an individual mouse (n = 8 per group). No significant differences were detected in any pairwise comparison for either measure. ns, not significant (P > 0.05); one-way ANOVA followed by Tukey's multiple-comparison test for all pairwise group comparisons.

Table S1. Primer sequences used for quantitative real-time PCR

Gene	Forward primer (5'-3')	Reverse primer (5'-3')
Aldoc	CACTCAATGCCTGGAGAGGACA	CGCCATCTCCACTGCCTTCATA
Pfkp	AAGAGGAAACCAAGCAGTGCGC	TTCCTCGGAGTTTCACGGCTTC
Pfkm	CTGTTCGCTCTACCGTGAGGAT	TTGGAACCACCTTGACCAGTCC
Slc2a12	CCAAATGCTGGACTGAGCCGAA	CTCACTAAGCACCAACCAAGGC
Slc2a13	GTCTGGCTTGTTGAGAAGGTGG	CGTTCTGACCTGACGGAGCTAT
Gapdh	GGTGAAGGTCGGTGTGAACG	CTCGCTCCTGGAAGATGGTG

Table S2. Summary statistics of genome alignment results

Sample	Total reads	Mapped reads	Mapped ratio (%)
SNI-1	46945270	44350039	94.47
SNI-2	60385878	59210354	98.05
SNI-3	53947358	52857584	97.98
Sham-1	53631412	52433856	97.77
Sham-2	59646130	58375024	97.87
Sham-3	59501862	58201257	97.81

Table S3. 126 metabolism-related DEGs from astrocyte-specific transcriptomic profiling

Gene	Sham 1	Sham 2	Sham 3	SNI 1	SNI 2	SNI 3	Log2FC	P value	Adj.P value
Cyp1b1	6.881986	6.728525	6.593499	2.5495	1.510633	1.050854	-1.98297	1.7265E-06	0.000114
Cyp2d22	83.30913	76.88213	87.52648	41.40081	48.86243	33.05997	-1.00625	0.002028589	0.02595
Cyp2j12	8.26082	9.424183	8.182254	20.06593	17.80036	21.28419	1.193263	8.46845E-09	1.55E-06
Cyp2j9	44.37406	42.73827	47.34488	20.98586	23.29105	19.82075	-1.0688	0.000465809	0.009026
Cyp2s1	1.427898	0.919868	0.672665	0.154726	0.553883	0.294351	-1.59049	0.002649149	0.031614
Cyp39a1	12.75878	10.59336	10.10038	6.432717	3.455768	3.154254	-1.35887	0.00177245	0.023461
Cyp4f14	14.6769	19.47398	21.55543	6.99047	11.33883	9.282553	-1.01255	0.003572226	0.038757
Cyp4f15	55.4388	63.14906	73.52872	19.07777	32.55825	20.13669	-1.42047	5.39997E-06	0.000273
Cyp4v3	7.022987	7.338958	7.667661	3.977907	2.970741	2.426717	-1.2325	0.000754923	0.012821
Cyp7b1	14.70051	15.73444	18.31353	7.760491	7.898835	8.569409	-1.00864	0.003686616	0.039546
Aldh1a2	7.055523	4.422579	2.982701	1.524921	2.120134	1.212135	-1.57395	0.000832357	0.013676
Chst8	0.550342	0.669633	0.74265	1.515695	1.180672	1.637617	1.14291	2.23663E-05	0.000851
Ugt8a	39.46668	61.37988	49.1722	77.0358	102.5673	128.8569	1.03994	2.00973E-06	0.00013
Sult4a1	0.913071	0.572069	1.010645	3.277535	1.595461	1.486624	1.349447	0.000436201	0.008609
Gldc	36.00597	49.48741	57.11006	14.57887	28.3958	24.29676	-1.08394	0.002044054	0.026102
Gpt2	37.33214	35.11909	35.72391	19.91962	17.99037	12.64173	-1.09754	0.001469571	0.020714
Got1l1	30.94053	16.86045	14.00998	4.594585	9.189264	9.242758	-1.42456	0.000694107	0.012032
Gpx8	48.7335	45.75879	42.01707	21.16807	17.82178	25.48823	-1.08212	0.001845781	0.024208
Mdh1b	17.07217	10.25482	6.675449	3.226576	5.308168	5.22473	-1.30521	0.003483326	0.038186
Eno4	10.47172	4.728451	4.453168	1.342897	3.267264	3.086251	-1.35252	0.002895302	0.033519
Pfkm	136.3064	152.6608	176.642	62.24305	90.00276	60.45913	-1.13027	0.000344994	0.007304
Pfkp	14.80732	13.41674	16.56449	5.251195	10.30607	6.677712	-1.0103	0.002617141	0.031338
Acot7	31.1536	33.33655	28.8074	65.60425	65.13534	62.58986	1.05115	1.94107E-08	3.1E-06
Acot11	24.84075	20.31597	21.71177	8.17216	14.58287	10.00244	-1.0295	0.001276847	0.018702
Acox2	1.784286	1.202354	1.071468	0.280179	0.548964	0.545483	-1.56177	0.0007062	0.012193
Agpat4	30.34388	32.78515	25.68687	68.69901	65.00756	67.05516	1.176594	1.67247E-09	4.94E-07
Pla2g7	146.5388	171.7899	191.8014	73.01854	88.86828	91.79105	-1.00787	0.001529033	0.021317
Plcb4	5.270958	3.669922	3.528856	1.697563	2.378472	1.894247	-1.06256	0.00163712	0.022295
Plcd4	21.05427	19.50127	20.79426	6.809586	11.45736	7.811084	-1.23422	4.10433E-05	0.001406
Plce1	7.69584	5.11539	6.13223	1.749639	2.685132	2.232373	-1.50656	3.10154E-07	2.88E-05
Pld2	20.46748	21.6766	23.9918	6.998604	13.64967	7.79068	-1.21756	0.000191645	0.00463
Pm20d1	1.394537	1.579346	2.022861	0.509329	0.980831	0.736408	-1.16617	0.004970126	0.049071
Smpdl3b	1.481481	2.179328	2.495329	0.648921	0.947653	0.771084	-1.37857	0.004999912	0.04923
Dio2	36.93732	52.62829	63.12881	13.63074	32.20363	22.61761	-1.15748	0.001722111	0.023058
Dio3	0.183052	0.202682	0.149311	0.016262	0	0	-5.0401	1.6304E-05	0.000667
Gstp1	136.7733	157.2937	135.9914	422.1206	296.8252	370.2328	1.340636	7.45402E-10	2.84E-07
Abca4	2.058692	2.801194	2.757771	0.360641	1.447605	0.723232	-1.58937	0.00024158	0.005618
Abcg1	13.65892	15.02019	13.7508	25.03733	32.72413	29.47432	1.039838	1.78737E-08	2.92E-06
Abcg4	2.777992	3.467387	3.391295	1.089579	2.050872	1.457054	-1.06768	0.002987553	0.034353
Slc1a3	210.337	265.1057	311.2742	82.40512	136.129	116.9749	-1.22949	0.000121694	0.003271
Slc1a5	7.01998	5.67338	5.658241	3.321021	2.719828	1.814537	-1.22415	0.001335442	0.019287
Slc2a12	12.22967	11.21396	10.56175	2.64077	5.559294	4.437637	-1.42803	1.03272E-05	0.000454
Slc2a13	1.730189	1.715657	1.755021	0.837582	0.77736	0.688453	-1.17499	0.002596372	0.031141
Slc4a4	59.7252	61.90745	74.70945	16.73068	32.87782	23.83345	-1.41869	5.11742E-06	0.000263

Gene	Sham 1	Sham 2	Sham 3	SNI 1	SNI 2	SNI 3	Log2FC	P value	Adj.P value
Slc4a5	4.694342	6.736431	7.554652	1.19446	3.231878	1.826573	-1.60229	2.42364E-05	0.000907
Slc4a10	7.924005	7.850864	8.56987	2.00304	4.824904	4.414727	-1.11463	0.001964574	0.025295
Slc6a11	295.079	283.162	365.6023	83.40576	211.2997	160.5124	-1.05199	0.003940983	0.041394
Slc6a15	2.374476	2.012972	1.412926	0.512674	0.926232	0.808087	-1.36815	0.002427907	0.029727
Slc6a17	1.354114	1.251722	1.562424	0.449418	0.878036	0.330174	-1.33032	0.001183407	0.01763
Slc7a10	78.50387	91.29792	110.2447	33.30354	65.51175	38.00063	-1.03343	0.003083426	0.035269
Slc7a11	12.55382	17.96344	19.93788	3.77528	7.672497	5.423209	-1.58046	1.60483E-06	0.000107
Slc8a1	3.617514	3.124261	2.655672	3.009232	9.848329	7.040555	1.082291	0.000330066	0.007141
Slc8a3	1.690134	1.804352	1.313145	3.073643	4.320423	3.925276	1.235392	2.09974E-08	3.28E-06
Slc13a3	13.34188	13.07932	13.68909	7.140331	5.39869	4.675515	-1.22035	0.000398298	0.008094
Slc13a4	11.3759	11.28985	11.29996	2.096544	5.289792	3.924414	-1.58638	1.8898E-06	0.000123
Slc13a5	20.78888	23.53457	27.34916	9.180242	13.79908	9.740564	-1.13125	0.000340318	0.007267
Slc16a3	0.424548	0.290109	0.318418	0.899079	0.988511	0.935195	1.450173	5.03101E-07	4.19E-05
Slc17a6	0.307185	0.293865	0.229606	0.615894	0.759252	1.536505	1.809515	8.22295E-06	0.000384
Slc24a5	1.556734	3.518092	2.557379	0.617271	0.732792	0.600472	-1.96823	4.25258E-05	0.001446
Slc26a7	1.60345	0.916339	0.91285	0.275812	0.362842	0.510632	-1.57858	0.000825682	0.013618
Slc27a6	0.875593	0.454003	0.342056	0.159422	0.172121	0	-2.33401	0.00451427	0.045649
Slc14a1	8.365906	11.51485	12.68079	3.70904	6.399929	5.881851	-1.02593	0.003749364	0.040095
Slc14a2	13.95888	7.825391	5.3713	1.508797	4.406167	4.167857	-1.42935	0.002505116	0.030351
Slc17a8	0.286754	0.429181	0.41354	0.084365	0.070844	0.130154	-1.98479	0.000473544	0.009111
Slc22a4	15.19234	11.68657	11.52339	3.577857	6.678049	4.739555	-1.35667	3.93783E-05	0.001362
Slc22a6	2.702216	1.961582	1.032196	0.224931	0.684658	0.495736	-2.01904	0.000106575	0.002931
Slc24a2	6.547903	8.045342	6.951471	9.526482	21.02805	21.9222	1.284344	1.57991E-07	1.71E-05
Slc24a4	8.562129	5.407005	3.777172	0.590056	2.883484	1.807891	-1.74852	0.000363939	0.007593
Slc29a4	11.2654	13.40292	15.08572	4.475915	6.709169	4.638276	-1.32905	5.4174E-05	0.001713
Slc30a2	1.001861	0.801346	0.798358	0.366789	0.093735	0.077325	-2.2741	0.000558937	0.010368
Slc30a3	0	0.039387	0.013195	0.031619	0.292053	0.153311	3.181308	0.000729794	0.012472
Slc34a3	0.187471	0.16605	0.041701	0.699758	0.545611	0.396587	2.054678	4.36872E-05	0.001471
Slc35f1	10.70829	9.428246	9.70068	4.009079	1.89483	1.80761	-1.95203	2.92243E-06	0.000171
Slc35g3	4.034292	2.120896	1.867888	0.699578	0.61681	0.863454	-1.87993	0.000321477	0.006993
Slc38a1	38.14938	8.156796	18.8193	1.200601	5.182344	10.17426	-1.97576	0.002835188	0.03302
Slc38a3	42.20707	39.38775	65.18712	23.26608	28.31828	19.06376	-1.05495	0.00335157	0.037222
Slc38a4	0.558104	0.604212	0.584901	0.158724	0.099958	0.03498	-2.57283	1.86409E-05	0.000738
Slc39a12	80.92702	84.39781	98.21225	22.44379	47.54874	40.95042	-1.24819	0.00012974	0.003437
Slc41a1	54.30164	52.21255	57.86508	18.59699	34.28578	26.21928	-1.05524	0.000816035	0.013494
Slc43a3	10.72022	6.659843	5.855861	2.279369	1.951822	1.167574	-2.10566	4.37831E-08	5.82E-06
Slc45a1	0.407887	0.273415	0.245236	0.576079	0.779899	0.77736	1.20319	0.000215304	0.005134
Slco1a5	1.215621	1.053632	0.988648	0.348505	0.409699	0.282665	-1.64615	0.000301198	0.006649
Nr1d1	107.9411	97.2779	104.1138	31.8377	59.43976	30.46196	-1.34536	2.9792E-05	0.001077
Nr1d2	29.52308	31.02756	30.42559	13.41776	13.5285	12.12811	-1.21927	4.6781E-05	0.00155
Nr2f1	12.79746	13.73359	14.26773	3.972631	8.806633	6.560969	-1.07692	0.001416773	0.020179
Nr2f2	17.93403	14.34991	14.42426	6.826271	5.14644	4.229429	-1.52749	6.5247E-06	0.000319
Ppargc1a	6.728996	6.105735	7.317699	1.129919	3.766956	2.597085	-1.42715	0.000200134	0.004814
Rxrg	1.679969	1.372753	1.923675	0.833325	0.548137	0.306108	-1.56015	0.003485396	0.038186
Ampd3	6.978147	6.883872	7.687002	2.437664	4.665775	3.350005	-1.04364	0.001644448	0.022366
Fmo1	17.41241	17.10173	17.09762	7.132336	5.482222	3.513196	-1.67815	2.01887E-06	0.00013

Gene	Sham 1	Sham 2	Sham 3	SNI 1	SNI 2	SNI 3	Log2FC	P value	Adj.P value
Taldo1	70.17234	88.17483	78.05254	186.9144	129.6945	158.0141	1.005554	1.5188E-06	0.000103
Bhmt	0.469946	0.392712	0.26827	0.075653	0.095285	0.18341	-1.67427	0.004325382	0.044317
Aass	6.024429	4.609695	5.644062	2.115523	2.982631	2.314055	-1.13496	0.001345154	0.019401
Hdc	14.95237	8.595896	6.224611	3.975274	4.61755	4.921111	-1.13955	0.003466227	0.038047
Gatm	136.7015	173.3542	155.9962	290.9953	312.7985	380.5347	1.078649	4.87112E-08	6.37E-06
Cdo1	17.51619	17.3899	16.88289	8.064106	7.641219	8.609518	-1.09081	0.001930062	0.024924
Igf1	2.321563	2.582152	2.335267	0.908545	0.339875	0.254339	-2.26817	9.67231E-06	0.000435
Acss3	3.533608	2.30685	2.15392	0.299635	1.170938	1.087125	-1.64414	0.00091829	0.014656
Adssl1	25.15309	28.98282	26.16146	65.01811	56.2763	57.55301	1.155305	6.58304E-09	1.32E-06
Phkg1	26.65278	30.50217	33.12419	10.34178	18.75551	10.60772	-1.18507	0.000305729	0.006721
Prkag3	0.509697	0.268744	0.367161	0.077656	0.0326	0.011413	-3.23508	8.30235E-06	0.000387
Rdh10	7.253188	6.840152	6.053972	2.245092	3.590491	3.868607	-1.05391	0.002138324	0.027004
Sphk1	0.750393	0.631626	0.584678	0.199073	0.103642	0.070203	-2.39884	1.92565E-06	0.000125
Aldoc	345.3783	427.056	500.3343	183.0465	229.3112	200.304	-1.05481	0.00106274	0.016236
Acsbg1	146.5379	173.077	202.0724	66.01183	102.7096	70.69392	-1.12367	0.000464677	0.009012
Acsf2	13.43832	13.97883	15.7292	5.47439	8.981597	6.620633	-1.03359	0.001410822	0.02012
Alas2	1.046106	0.588343	0.384168	5.206336	0.490715	1.88905	1.909991	0.001050748	0.016114
Ampd2	8.710465	7.473338	10.38526	2.605026	5.044274	4.41867	-1.13856	0.000641991	0.011356
Bbox1	12.67899	7.289756	6.360531	2.128771	4.144306	4.068017	-1.34828	0.000462668	0.008989
Eci3	1.238845	1.48289	1.430099	0.571338	0.449769	0.157411	-1.81678	0.002768781	0.032491
Elovl1	11.38333	13.67747	13.01071	29.65201	28.1926	19.15972	1.016228	1.22153E-06	8.79E-05
Elovl2	9.937165	11.77113	14.84921	3.591185	6.284137	5.402147	-1.25876	0.000119723	0.00323
Fads2	268.3005	253.9306	291.165	74.57411	143.7401	113.3912	-1.29406	2.47488E-05	0.00092
Fmo4	0.864763	0.862854	0.999473	0.33922	0.311553	0.224298	-1.63989	0.000407091	0.008187
Fmod	12.52612	8.263735	5.624829	2.107658	3.995086	3.142412	-1.51457	9.44562E-05	0.002678
Gad2	0.079735	0.156957	0.165554	0.415739	0.277693	0.299874	1.304158	0.001404884	0.020078
Glt1d1	0.046289	0.081999	0.154459	0.357899	0.248714	0.402563	1.835595	0.000189113	0.00459
Me3	1.026636	0.912398	0.922613	0.514814	0.386627	0.28766	-1.26697	0.003686801	0.039546
Med12l	1.978641	2.190214	2.22505	0.348224	1.586988	0.697956	-1.2799	0.004920618	0.048701
Mertk	57.60571	41.08006	48.52974	16.04471	34.69006	15.56699	-1.15081	0.001631283	0.022271
Mest	14.96545	15.2288	13.59038	6.73276	2.316473	2.227629	-1.95706	6.46136E-05	0.001974
Nme5	25.17182	18.11795	12.35978	6.094062	6.954201	9.450228	-1.30654	0.000692825	0.01202
Nme9	20.47539	11.61678	9.124184	2.571017	5.296501	6.074569	-1.56377	0.00039935	0.008097
Pank2	3.940493	1.363168	3.643814	0.783701	1.273235	0.991314	-1.5535	0.000384838	0.007923
Pygm	33.80279	29.78788	37.12056	14.87106	20.19825	13.76594	-1.04423	0.001035271	0.01596
Scd3	1.345846	1.842387	1.279234	5.137749	4.108758	5.852369	1.756912	9.90777E-10	3.4E-07

Table S4. Node centrality measures of the protein–protein interaction network of metabolism-related DEGs.

Gene symbol	Degree	Betweenness centrality	Closeness centrality
Slc1a3	32	1631.411291	0.003424658
Ppargc1a	24	2755.116026	0.003731343
Gad2	20	1446.914609	0.003496503
Pygm	18	812.5064103	0.003267974
Adssl1	16	411.8948718	0.002915452
Eno4	16	237.4237179	0.00286533
Taldo1	16	239.6276991	0.002898551
Slc1a5	16	226.2	0.002923977
Gldc	14	439.9180162	0.002881844
Cyp7b1	14	2594.366667	0.002688172
Pfkm	14	40.59038462	0.002857143
Gpx8	14	3347.463889	0.003759398
Acox2	12	808	0.002247191
Aldoc	12	225.2910256	0.00295858
Pfkp	12	67.68026316	0.002824859
Cyp1b1	12	999.3	0.003012048
Cyp2s1	10	184.2333333	0.002849003
Cyp4f14	10	1543.933333	0.002985075
Cyp2j9	10	1036	0.003355705
Slc7a11	10	656.8057644	0.003389831
Slco1a5	8	1106	0.002267574
Got1l1	8	218.3440733	0.003125
Cdo1	8	246.6666667	0.002762431
Cyp4f15	8	35.66666667	0.002840909
Cyp2d22	8	51.73333333	0.002570694
Igf1	8	598.7916667	0.003508772
Slc38a4	8	169.9029866	0.002717391
Slc16a3	8	72.3409421	0.002793296
Slc38a1	8	169.9029866	0.002967359
Slc38a3	8	24.45795739	0.00295858
Gatm	6	67.61403509	0.002785515
Cyp39a1	6	8.133333333	0.002840909

Gene symbol	Degree	Betweenness centrality	Closeness centrality
Gstp1	6	1166.3	0.003344482
Cyp2j12	6	779.5666667	0.003289474
Fads2	6	6	0.333333333
Mdh1b	6	19.66666667	0.002409639
Slc17a8	6	0	0.00286533
Slc6a11	6	18.78830409	0.002873563
Slc17a6	6	0	0.00286533
Gpt2	6	351.6660819	0.00330033
Phkg1	6	1	0.002583979
Prkag3	6	46.5	0.00297619
Slc22a6	6	490	0.001930502
Slc4a4	6	166	0.002710027
Slc7a10	6	3.902986633	0.002710027
Mertk	4	166	0.001879699
Abcg1	4	328	0.002217295
Acot11	4	166	0.001915709
Acsf2	4	0	0.001897533
Acss3	4	0	0.001897533
Ampd3	4	0	0.002352941
Ampd2	4	0	0.002352941
Pld2	4	2	0.5
Alas2	4	0	0.002331002
Aldh1a2	4	1.5	0.002314815
Rdh10	4	14.33333333	0.002421308
Bhmt	4	10.64102564	0.002364066
Dio2	4	166	0.00286533
Nme5	4	0	0.002347418
Rxrg	4	166	0.00286533
Nr1d1	4	0	0.002857143
Nr1d2	4	0	0.002857143
Nr2f1	4	0	0.002857143
Nr2f2	4	0	0.002857143
Plcb4	4	0	0.5
Plcd4	4	0	0.5

Gene symbol	Degree	Betweenness centrality	Closeness centrality
Plce1	4	0	0.5
Slc13a4	4	166	0.001669449
Slc2a12	4	3.902986633	0.002695418
Slc30a2	4	0	0.5
Slc30a3	4	0	0.5
Slc39a12	4	0	0.5
Aass	2	0	0.002325581
Abca4	2	0	0.001626016
Acot7	2	0	0.001652893
Eci3	2	0	0.001893939
Slc27a6	2	0	0.001893939
Acsbg1	2	0	0.001893939
Agpat4	2	0	0.333333333
Bbox1	2	0	1
Cyp4v3	2	0	1
Fmo1	2	0	0.002247191
Hdc	2	0	0.002197802
Dio3	2	0	0.002314815
Elovl1	2	0	0.2
Elovl2	2	0	0.2
Scd3	2	0	0.2
Fmod	2	0	0.002717391
Me3	2	0	0.002336449
Med12l	2	0	0.002314815
Mest	2	0	0.002217295
Pla2g7	2	0	0.002666667
Sphk1	2	0	0.333333333
Pm20d1	2	0	1
Slc41a1	2	0	1
Slc14a1	2	0	0.002849003
Slc13a3	2	0	0.001663894
Slc4a5	2	0	0.001466276
Slc2a13	2	0	0.002666667
Slc22a4	2	0	0.001908397

Gene symbol	Degree	Betweenness centrality	Closeness centrality
Slc26a7	2	0	0.002212389
Slc6a15	2	0	0.002380952
Slc8a1	2	0	1
Slc8a3	2	0	1

Note. The protein–protein interaction (PPI) network was constructed using STRING, with the 126 metabolism-related differentially expressed genes as input. Node degree, betweenness centrality, and closeness centrality were calculated using the CentiScaPe plugin in Cytoscape.
